# FaesAP3_1 Regulates the *FaesELF3* Gene Involved in Filament-Length Determination of Long-Homostyle *Fagopyrum esculentum*

**DOI:** 10.3390/ijms232214403

**Published:** 2022-11-19

**Authors:** Zhiyuan Ma, Qingyu Yang, Lingtian Zeng, Jiayi Li, Xinyu Jiao, Zhixiong Liu

**Affiliations:** College of Horticulture and Gardening, Yangtze University, Jingzhou 434025, China

**Keywords:** common buckwheat, filament-length determination, floral organ identity genes, stamen, target genes

## Abstract

The identification downstream genes of floral organ identity regulators are critical to revealing the molecular mechanisms underlying floral morphogenesis. However, a general regulatory pathway between floral organ identity genes and their downstream targets is still unclear because of the lack of studies in nonmodel species. Here, we screened a direct downstream target gene, *FaesELF3*, of a stamen identity transcription factor, FaesAP3_1, in long-homostyle (LH) *Fagopyrum esculentum* moench by using yeast one-hybrid (Y1H) and dual-luciferase reporter (DR) assays. Furthermore, *FaesAP3_1*-silenced LH plants that produced flowers with part stamens or anthers homeotically converted into a tepaloid structure, and *FaesELF3*-silenced plants that had flowers with part stamens consisting of a short filament and empty anther (male sterile anther). All these suggested that transcription factor (TF) FaesAP3_1 directly activates *FaesELF3* in order to regulate filament elongation and pollen grain development in LH buckwheat. Our data also suggested that other stamen development pathways independent of *FaesAP3_1* remain in *F. esculentum.*

## 1. Introduction

Common buckwheat (*Fagopyrum esculentum*) grains are gluten-free, but are rich in bioactive compounds (such as rutin, quercetin and polysaccharides, etc.) and lysine, which make it a popular functional food with health benefits; subsequently, demand has increased in recent years [1,2]. However, *F. esculentum* is a heteromorphic self-incompatibility (HSI) crop due to its distylous flowers (pin and thrum). In pin flowers, a long style is combined with short stamens and small pollen grains; in thrum flowers, a short style is combined with long stamens and large pollen grains. The pin plants and thrum plants are equal in the population. In addition, obligate outcrossing pollinations occur strictly between pin and thrum plants, which results in low achene-set-rate and yield [3,4]. Improving the yield stability of *F. esculentum* requires a better understanding of the molecular basis of the HIS or developing a self-compatible (SC) common buckwheat line. Previous studies have suggested that buckwheat HIS was determined by a single genetic *S* locus resulting in heterozygous dominant (*S/s*) thrum plants and homozygous recessive (*s/s*) pin plants [5]. A buckwheat *EARLY FLOWRING3* homologue (*S-ELF3*) has been identified as a candidate *S* locus gene and is present only in thrum plants, and determines style length [6,7].

*Arabidopsis ELF3,* a component of the circadian evening complex (EC), played a crucial role in inhibiting hypocotyl elongation, flowering, and integrating environmental light and ambient temperature signals of the circadian clock [8,9]. Previous studies have suggested that soybean ELF3 ortholog interacts with LUX (LUX ARRYTHMO) to form EC in order to control photoperiod sensitivity and adapt to regulate flowering during short days [10]. Monocot *ELF3* orthologs, *BdELF3* from *Brachypodium distachyon,* and *SvELF3* from *Setaria viridis* showed function conservation and were capable of rescuing hypocotyl elongation, flowering time, and arrhythmic clock phenotypes of *Arabidopsis elf3* [11]. However, two rice *ELF3* homologs, *OsELF3–1* and *OsELF3–2*, exhibited function overlap and divergence after gene duplication events. Both genes exhibited shared functions in the regulation of rice circadian clocks. However, *OsELF3-2* was also identified as a negative regulator of immunity against *Magnaporthe oryzae*, while *OsELF3-2* had a unique role involved in the suppression of phytochrome signaling to regulate rice flowering [12,13]. Although the ELF3 homologs interacting with other EC component to regulate flowering and flower development have been explored in several model plants, the molecular mechanisms and how they are regulated by upstream genes have not yet been elucidated.

**Figure 1:**
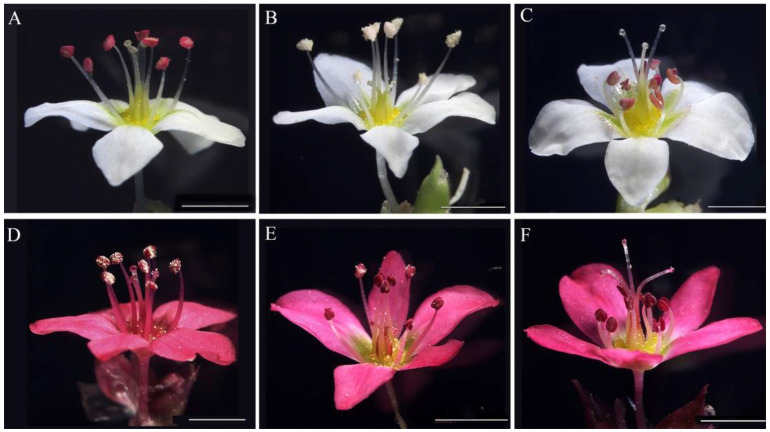
Distylous flower and long-homostyle flower of *F. esculentum*. (**A**) White long-homostyle flower with long style and long stamens; (**B**) White thrum flower with short style and long stamens; (**C**) White pin flower with long style and short stamens; (**D**) Red LH flower of daughter lines between white LH flower plant and red flower common buckwheat; (**E**) Red thrum flower with short style and long stamens; (**F**) Red pin flower with long style and short stamens. Scale bar = 2 mm.

In this study, we have developed a self-compatible common buckwheat line with uniform long-homostyle (LH) flowers that could freely outcross with pin, thrum or LH plants (Figure 1). Moreover, another *ELF3* homologue (*FaesELF3*) and its promoter (*pFaesELF3*) were separately isolated from LH plants. Further study suggested that two key CArG-box motifs for the MADS-box transcription factor (TF) recognition and binding are located at the *FaesELF3* promoter region [14,15]. MADS-box genes encoding key regulators are involved in virtually every aspect of plant reproductive development. How different MADS-box transcription factors work together to regulate flowering time control, inflorescence architecture, floral organ identity determination, and seed development are well studied [16]. However, their downstream target genes specifying floral organ identity remain unclear. To further elucidate whether or not *FaesELF3* was a candidate target gene of buckwheat MADS-box TF, three B-class MADS genes, *FaesAP3_1, FaesAP3_2* and *FaesPI* [4,17], and a C-class MADS-box gene, *FaesAG* [18], previously identified by us, were screened. The regulatory relationship between *FaesAP3_1* and *FaesELF3* was confirmed by yeast one-hybrid (Y1H) and dual-luciferase reporter assays (DR), respectively. Furthermore, the functions of both genes involved in floral development were revealed by VIGS. Our study confirms a downstream target of B-function MADS-box TF in buckwheat. We thus revealed new insights into the interactions of B-class MADS-box TF and *ELF3* ortholog in filament-length determination and anther development in *F. esculentum*.

## 2. Results

### 2.1. Isolation and Characterization of FaesELF3 and FaesELF3 Promoter (pFaesELF3) from Long-Homostyle Common Buckwheat 

The 2364 bp *FaesELF3* cDNA contains a 2076 bp ORF (Open Reading Frame, ORF) encoding 691 amino acids (aa) (Genbank accession number: OP572281.1). Phylogenetic tree analysis indicated that *FaesELF3* was an *EARLY FLOWERING 3* (*ELF3*) orthologous gene. The gene was designated as *FaesELF3* (*Fagopyrum esculentumELF3*) (Supplementary Figure S1).

A 1849 bp *FaesELF3* promoter *(pFaesELF3)* (−1278/+571) (Genbank accession number: OP572282.1) was cloned from LH common buckwheat. The putative cis-acting elements and transcription start site (TSS) of *pFaesELF3* was displayed in Figure S2 (Supplementary Figure S2). The *pFaesELF3* contains three CONSTANS protein binding sites (CCAATBOX1) associated with flowering [19]. In addition, the *pFaesELF3* contains two key CArG-box motifs (−1099/−1090 and +236/+245) for MADS-box TF recognition and binding [14]. Moreover, *pFaesELF3* contains six AACAAA-/TTTGTT-motifs for floral homeotic protein APETALA2 recognition and action [20]. In addition, *pFaesELF3* has three POLLEN1LELAT52-boxes and five GTGANTG10-boxes, two cis-regulatory elements within the promoter of stamen-development genes [21,22]. All these data suggested that *pFaesELF3* may drive the *FaesELF3* gene to regulate flowering and stamen development. Moreover, a petal epidermis-specific MYB transcription factor binding site (MYBCORE-box) [23], as well as two embryo-specific and ABA–responsive elements (DPBFCOREDCDC3-boxes) [24], were located at *pFaesELF3*, which suggested that the *FaesELF3* expression may extend to perianth, pistil and fruits. In addition, some gibberellin-responsive elements (WRKY71OS-box, TATCCAOSAMY-box and PYRIMIDINEBOXOSRAMY1A-box), an auxin-responsive element (NTBBF1ARROLB-box) and a cytokinin-responsive element (ARR1AT-box) were also found at *pFaesELF3* [25,26,27,28,29]. All above data suggested that abscisic acid (ABA), gibberellin (GA), auxin (IAA) and cytokinin (CK) signaling pathways may also directly regulate *FaesELF3* expression. Furthermore, two MYCCONSENSUSAT-boxes and an ACGTATERD1-box were located at *pFaesELF3*, which indicates that *FaesELF3* expression could be induced by freezing, dehydration stress and dark [30,31]. Moreover, a CBFHV-motif for DREB/CBF transcription factor recognition and action is also found in the *pFaesELF3* region [32]. All these data suggested that *pFaesELF3* may drive corresponding genes to regulate flowering and floral organ development in different pathways. 

### 2.2. Deletion Analysis of the pFaesELF3 in Transgenic Arabidopsis

**Figure 2:**
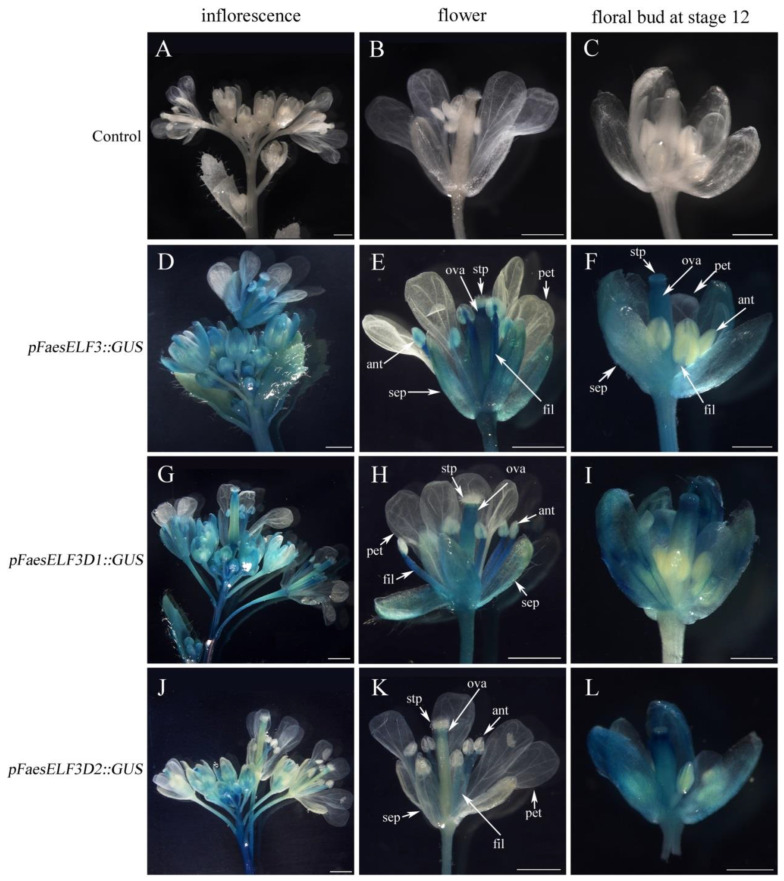
Histochemical GUS staining in the T1 generation of *pFaesELF3*::*GUS* transgenic *Arabidopsis* and deletion analysis of the *pFaesELF3* promoter. (**A**) Wild-type *Arabidopsis* inflorescence; (**B**) Wild-type *Arabidopsis* flower; (**C**) Stage 12 floral bud of wild-type *Arabidopsis* [33]; (**D**) Inflorescence of *pFaesELF3*::*GUS* transgenic *Arabidopsis*; (**E**) Flower of *pFaesELF3*::*GUS* transgenic *Arabidopsis*; (**F**) Stage 12 floral bud of *pFaesELF3*::*GUS* transgenic *Arabidopsis*; (**G**) Inflorescence of *pFaesELF3D1*::*GUS* transgenic *Arabidopsis*; (**H**) Flower of *pFaesELF3D1*::*GUS* transgenic *Arabidopsis*; (**I**) Stage 12 floral bud of *pFaesELF3D1*::*GUS* transgenic *Arabidopsis*; (**J**) Inflorescence of *pFaesELF3D2*::*GUS* transgenic *Arabidopsis*; (**K**) flower of *pFaesELF3D2*::*GUS* transgenic *Arabidopsis*; (**L**) Stage 12 floral bud of *pFaesELF3D2*::*GUS* transgenic *Arabidopsis*. sepal (sep), petal (pet), anther (ant), filament (fil), ovary (ova), stigmatic papillae (stp); scale Bars: (**A**,**B**,**D**,**E**,**G**,**H**,**J**,**K**) 1 mm; (**C**,**F**,**I**,**L**) 500 μm.

A series of *5′* deletion fragments of *pFaesELF3* were separately fused to the *GUS* gene and transformed into *Arabidopsis* to analyze the regulatory effect of different promoter regions. *Beta-glucuronidase* (GUS) staining was separately examined in the T1 generation of *pFaesELF3*::*GUS*, *pFaesELF3D1*::*GUS* and *pFaesELF3D2*::*GUS* independent transgenic lines (Figure 2). GUS staining suggested that *pFaesELF3*(−1278/+571) and *pFaesELF3D1* (−892/+571) constructs presented similar expression patterns. GUS staining was detected in the inflorescence, sepal, filament and ovary of mature flowers, but was absent in the petals and anthers (Figure 2E,H). In addition, *GUS* expression was clearly observed in the early development floral buds from initiation until stage 12, where sepal, ovary, stigma and stigmatic papillae were intensive, but was absent in the petals and stamens (filaments and anthers) of *pFaesELF3D2*::*GUS* (+246/+571) transgenic *Arabidopsis* (Figure 2K). However, only weak *GUS* staining was observed in the filaments and ovaries of mature flowers in *pFaesELF3D2*::*GUS* transgenic *Arabidopsis* (Figure 2L). These data suggested that the *pFaesELF3* may drive *FaesELF3* to be involved in regulating filament elongation and pistil development in *F. esculentum*. 

### 2.3. B-Class MADS-Box Transcription Factor FaesAP3_1 to Target FaesELF3 for Stamen Development

**Figure 3:**
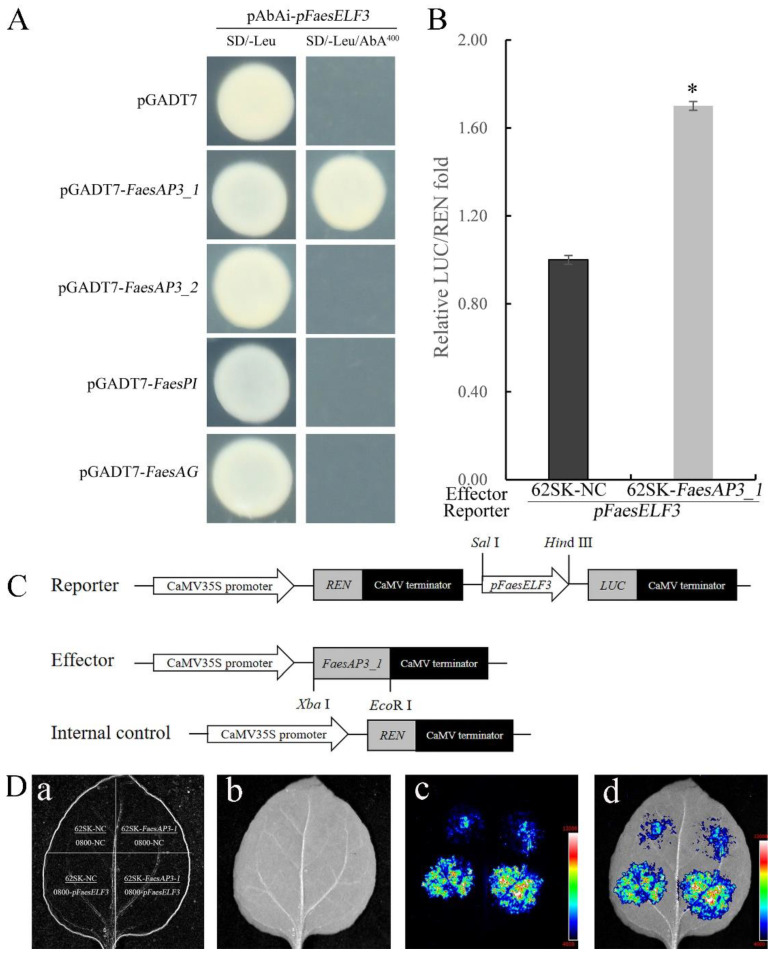
Positive regulation of *FaesELF3* by B-class MADS-box transcription factor FaesAP3_1. (**A**) Y1H screens which MADS-box proteins could directly bind to the *pFaesELF3* region containing CArG-box. pGADT7 vector was used as negative control; (**B**) Relative reporter activity (LUC/REN) in tobacco leaves of transient expression; (**C**) Schematic structure of the reporter and effecter constructs; (**D**) Luciferase signals (LUC/REN) were captured in *N. benthamiana* leaf cells; (**a**): diagram of reporter and effector constructs; (**b**): bright field image; (**c**): dark field image; (**d**): merge image. Asterisk (*) indicates a significant difference (*p* < 0.05 by Student’s *t*-test).

Previous studies suggest that MADS-box transcription factors bind to CArG-box to regulate the expression of target genes involved in floral development [15]. To screen which MADS-box transcription factors (FaesAP3_1, FaesAP3_2, FaesPI or FaesAG) directly regulated *FaesELF3* in common buckwheat, yeast one-hybrid (Y1H) and dual-luciferase reporter assays (DR) were performed. Yeast one-hybrid (Y1H) assays showed that only B-class MADS-box transcription factor FaesAP3_1 could bind to the target fragment of the *FaesELF3* promoter region to activate reporter gene expression (Figure 3A). In addition, the regulatory effect on gene expression was further evaluated using the dual-luciferase reporter assay (DR) in *N. benthamiana* leaf protoplasts. Transient expression assay further showed that the positive regulation of *FaesAP3_1* to *FaesELF3* in the plants (Figure 3B–D). The dual-luciferase reporter assays further showed that when the reporter construct carried the *pFaesELF3* promoter region, *LUC* expression was considerably upregulated (*p* < 0.05) (Figure 3B,D).

### 2.4. Expression Analysis of FaesELF3 in LH Flower F. Esculentum

**Figure 4:**
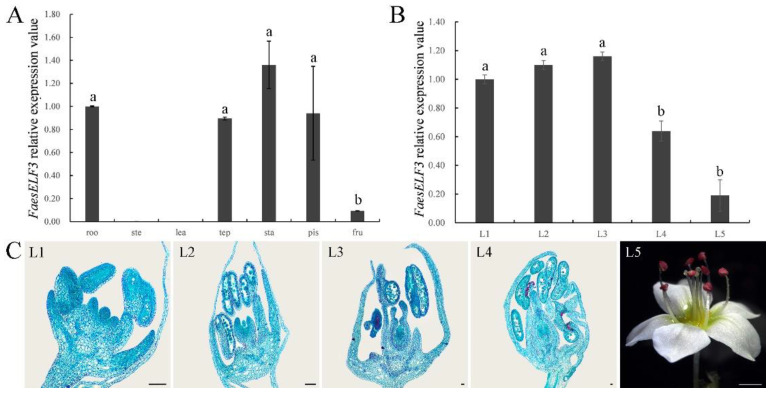
Morphology, *FaesELF3* expression in different organs and floral buds at sequential development stage of long-homostyle *F. esculentum*. (**A**) *FaesELF3* expression in the root (roo), stem (ste), juvenile leaf (lea), tepal (tep), stamen (sta), pistil (pis), and 6-day-old fruit (fru) were detected by qRT-PCR in LH *F. esculentum*; (**B**) *FaesELF3* expression was detected by qRT-PCR in LH floral buds at sequential development stage; (**C**) Cytomorphological section of LH floral buds at sequential development stages; L1: pistil primordium appearance and anther at microspore mother cell stage; L2: filament rapidly elongating and anther at microspore tetrads stage; L3: anther at mononuclear microspore; L4: mature floral bud with mature pollen before bloom; L5: flower in bloom. Scale bar: (L1–L4) 100 μm; (L5) 1 mm. Different letters indicate significant difference (*p* < 0.05 by *LSD*).

*FaesELF3* was obviously expressed in roots, tepals, stamens, pistils and young fruits of the LH flower *F. esculentum*, but the *FaesELF3* transcript was absent in the stems and leaves. (Figure 4A). In addition, the expression level of *FaesELF3* in tepals, stamens and pistils was significantly higher than that of the young fruits (*p* < 0.05, LSD). Obvious expression of *FaesELF3* was detected at pistil primordium emergence and when the anthers were at microspore mother cell stage in LH floral buds (Figure 4B,C). In addition, *FaesELF3* expression increased constantly during the filament rapid elongating stage and achieved its peak until the anthers were at mononuclear microspore stage (Figure 4B,C (L3)). Then, the *FaesELF3* expression began to drop sharply from floral buds with anthers at mature pollen stage to blossom (*p* < 0.05, LSD) (Figure 4B,C (L4,L5)).

### 2.5. Characterization of FaesAP3_1 and FaesELF3-Silenced Plants

**Figure 5:**
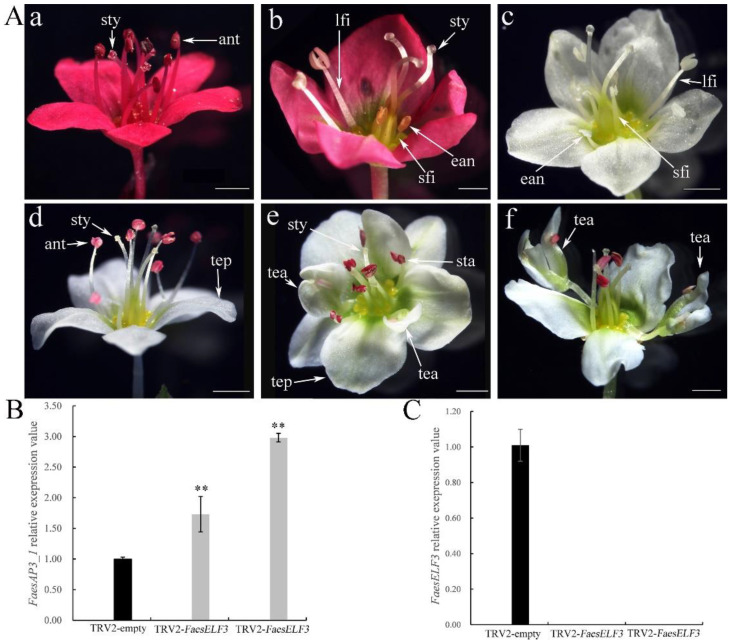
Phenotypes of VIGS-silenced *F. esculentum* LH flowers, as well as *FaesELF3* and *FaesAP3_1* expression in TRV2-empty and TRV2-*FaesELF3*-treated LH flowers with strong phenotypic changes. (**A**) Phenotypes of VIGS-treated *F. esculentum* LH flowers; (**a**): TRV2-empty-treated red LH flower with three long styles and eight long stamens; (**b**): TRV2-*FaesELF3*-treated red LH flower with three long styles, two long stamens and three short stamens consisting of short filaments and empty anthers (male sterile anther); (**c**): TRV2-*FaesELF3*-treated white LH flower with three long styles, three long stamens and five short stamens consisting of short filaments and empty anthers (male sterile anther); (**d**): TRV2-empty white LH flower with five tepals, eight long stamens and three long styles; (**e**): TRV2-*FaesAP3_1*-treated white LH flower with six normal tepals, two tepals with anther attachments, six long stamens and three long styles; (**f**): TRV2-*FaesAP3_1*-treated white LH flower with three tepals, two filaments with anthers homeotically converted into tepaloid structures (tepal attachment of anther) on the top, four stamens and three long styles. (**B**) *FaesAP3_1* expression was separately detected by qRT-PCR in TRV2-empty and TRV2-*FaesELF3*-treated LH flowers; (**C**) *FaesELF3* expression was separately detected by qRT-PCR in TRV2-empty and TRV2-*FaesELF3*-treated LH flowers. tepal (tep), stamen (sta), style (sty), long filament (lfi), short filament (sfi), empty anther (ean), tepals attachment of anther (tea); scale bar = 1 mm. Double asterisk (**) indicates a statistical significance at *p*-value ≤ 0.01 level.

To uncover the functions of *FaesAP3_1* and *FaesELF3*, a virus-induced gene silencing (VIGS) technique via the Tobacco Rattle Virus (TRV) system was used to knock down their expression in LH *F. esculentum*. TRV2-*FaesELF3*-treated LH plants with strong phenotypic changes and produced flowers with part of stamens consisting of short filaments and empty anthers (male sterile anther) (Figure 5A(b,c)). Moreover, a range of phenotypes was observed in the *FaesAP3-1*-silenced flowers. Some flowers had increased tepal numbers and reduced stamen numbers with part stamens homeotically converted into tepaloid structures (tepal attachment of anther) (Figure 5A(e)). Other flowers had reduced stamens with anthers homeotically converted into tepaloid structures (tepal attachment of anther) on the top of the filaments (Figure 5A(f)). In addition, *FaesAP3_1* and *FaesELF3* expression in *FaesELF3*-silenced plants were separately detected by qRT-PCR. *FaesELF3* expression was not detected in the strong phenotypic flowers of the TRV2-*FaesELF3*-treated LH plants (Figure 5A(b,c),C), while *FaesAP3_1* expression upregulated significantly in the flowers of TRV2-*FaesELF3*-treated LH plants (*LSD, p* < *0.01*) (Figure 5A(b,c),B). Our previous study suggested that *FaesAP3_1* was exclusively expressed in the stamen and was involved only in stamen development in *F. esculentum* [34]. Interestingly, some abnormal stamens were still observed even when *FaesAP3_1* expression upregulated significantly in TRV2-*FaesELF3*-treated LH buckwheat (Figure 5A(b,c),C).

## 3. Discussion

Previous studies have suggested that *Arabidopsis* B-class MADS-box genes, *APETALA3* (*AP3*) and *PISTILLATA* (*PI*) are expressed in petals and stamens, and work together to control the formation of petals and stamens during *Arabidopsis* flower development [35]. Moreover, AP3/PI may act as a bifunctional TF switch between the activation of male and the repression of female development [35]. The identification downstream genes of floral organ identity regulators are critical to understanding the mechanisms underlying floral morphogenesis. Yet, a general picture of the regulatory pathway between floral organ identity genes and their targets is still lacking because of the lack of studies in nonmodel species [36,37]. In *F. esculentum,* two *AP3* orthologs, *FaesAP3_1* and *FaesAP3_2*, resulted from a gene duplication event; they showed shared functions in determining stamen filament identity and worked together to determine normal stamen development [4,34]. *FaesAP3_1* was expressed only in stamens and exclusively required for stamen formation, while *FaesAP3_2* was expressed in petaloid tepal, stamens and pistils, and was mainly involved in regulating stamen development [34]. However, the downstream targets genes for *FaesAP3_1* and *FaesAP3_2* remain unclear during buckwheat stamen development. In this study, we proved that *FaesELF3* is a direct downstream target of FaesAP3_1, and is involved in filament-length determination and pollen grain development. *FaesELF3* didn’t interact with other B function TFs, such as FaesAP3_2 and FaesPI in distylous *F. esculentum*. Furthermore, The *FaesAP3_1* function controlling stamen development was further assayed by the VIGS silenced method. Results showed that TRV2-*FaesAP3_1*-treated LH buckwheat produced flowers with part stamens or anthers homeotically converted into tepaloid structures. Interestingly, only part abnormal stamens were observed even when *FaesAP3_1* expression upregulated significantly in TRV2-*FaesELF3*-treated LH buckwheat. These data suggest that *FaesELF3* is a key direct downstream target of FaesAP3_1 involved in normal stamen development; on the other hand, another stamen development pathway independent of *FaesAP3_1* remains in *F. esculentum*.

## 4. Materials and Methods

### 4.1. Plant Material and Growth Conditions

Long-homostyle (LH) common buckwheat was planted under natural conditions in Jingzhou, Hubei Provence, China. The roots, stems, juvenile leaves, tepals, stamens, pistils and 6-day-old fruits (achenes) of LH plants were dissected, and were immediately frozen and stored in liquid nitrogen. Moreover, the floral buds at sequential developmental stages were sampled from LH plants. Each sample was divided in half; one half was immediately frozen in liquid nitrogen until used, and the other was fixed in FAA [38% formaldehyde: acetic acid: 70% ethanol = 1:1:18 (*v*/*v*)]. The *Arabidopsis Col−0* (Columbia ecotype) seeds were obtained from the *Arabidopsis* Biological Resource Center (ABRC) at Ohio State University, USA. *Nicotiana benthamiana* for dual-luciferase reporter assay were grown in the growth chamber at 22 °C under long-day conditions (16 h light, 8 h dark).

### 4.2. Isolation and Characterization of FaesELF3 and FaesELF3 Promoter (pFaesELF3) from Long-Homostyle Common Buckwheat

Total RNA was extracted from LH buckwheat floral buds using an EASY spin Plant RNA Kit (Aidlab, Beijing, China), referring to the manufacturer’s protocol. The 3′ end and 5′ partial cDNA sequences of *FaesELF3* were obtained by using the 3′-full RACE Core Set Ver. 2.0 kit (TaKaRa, Shiga, Japan) with gene-specific primer 3RELF3GSP, and 5′ Full-RACE Kit (TaKaRa, Shiga, Japan) with gene-specific primers 5RELF3GSP1, 5RELF3GSP2 and 5RELF3GSP3 (Supplementary Table S1), based on the manufacturer’s protocol. Phylogenetic analysis of *FaesELF3* was referenced in You et al. [2]. Putative FaesELF3 protein sequences and other ELF3 homolog proteins from different species and were selected from phylogenetic trees from NCBI Genbank (Supplementary Table S2).

Buckwheat genomic DNA was extracted from LH buckwheat juvenile leaves using the CTAB Plant Genomic DNA Rapid Extraction Kit (Aidlab, Beijing, China), according to the manufacturer’s protocol. The *FaesELF3* 5′ flanking region was isolated from LH buckwheat genomic DNA by using the Genome Walking Kit (TaKaRa, Shiga, Japan), following the manufacturer’s protocol. The gene-specific primers DpELF3SP1, DpELF3SP2 and DpELF3SP3 (Supplementary Table S1) were used for amplifying the first walking sequencing. The gene-specific primers DpELF3SP4, DpELF3SP5, and DpELF3SP6 (Supplementary Table S1) were used for amplifying the second walking sequencing. The putative transcription start site of *FaesELF3* was searched through 5′RACE according to the above method. The cis-acting elements located at the *FaesELF3* promoter region were searched in the PLACE database [38].

### 4.3. Characterization of pFaesELF3 Activity from the 5′ Deleted Promoter Fragments in Transgenic Arabidopsis

Three 5′-deletion fragments of *pFaesELF3* were separately cloned into the pCAMBIA1300 vector by using the ClonExpress^®^ Ultra One Step Cloning Kit (Vazyme, Nanjing, China), following the manufacturer’s protocol. The *pFaesELF3* (−1278/+571) was amplified with the primers TpFaesELF3F and TpFaesELF3R. The *pFaesELF3D1* (−892/+571) was amplified with the primers TpFaesELF3F1 and TpFaesELF3R. The *pFaesELF3D2* (+246/+571) was amplified with the primers TpFaesELF3F2 and TpFaesELF3R. Then, three constructs were separately transformed into *Col−0 A. thaliana* using the floral-dip method suggested by Clough and Bent [39]. Transgenic *Arabidopsis* seedlings were screened, cultivated and prepared for histochemical *GUS* staining with the method suggested by Liu et al. [40]. The *GUS* staining samples observed and the photomicrographs taken referred to the method described by You et al. [2].

### 4.4. Yeast One-Hybrid Assay

Yeast one-hybrid (Y1H) assay was performed using the Yeast One-Hybrid Media Kit (Coolaber, Beijing, China). The *pFaesELF3* region (−43/+571) containing a CArG-box motif was cloned into pAbAi plasmid to construct pBait-AbAi vectors with the primers Y1HpFaesELF3F and the reversed primer Y1HpFaesELF3R (Supplementary Table S1). Four common buckwheat MADS-box gene (*FaesAP3_1*, *FaesAP3_2*, *FaesPI* and *FaesAG*) cDNAs containing full-length ORFs (Open Reading Frame) were separately cloned into pGADT7 plasmid to construct prey vectors, but with the primers Y1HFaesAP3_1F and Y1HFaesAP3_1R for *FaesAP3_1*, Y1HFaesAP3_2F and Y1HFaesAP3_2R for *FaesAP3_2*, Y1HFaesPIF and Y1HFaesPIR for *FaesPI*, as well as Y1HFaesAGF and Y1HFaesAGR for *FaesAG* (Supplementary Table S1). The linearized plasmid pAbAi-*pFaesELF3* was transformed into the Y1H Gold competent cells to generate bait-reporter strains. The transformants were selected on SD/-Ura media and screened for minimal inhibitory concentration of Aureobasidin A (AbA) for the bait-reporter strains. Four prey plasmids pGADT7-*FaesAP3_1*, pGADT7-*FaesAP3_2*, pGADT7-*FaesPI* and pGADT7-*FaesAG* were separately transformed into the bait yeast strains for yeast one-hybrid assays according to the manufacturer’s protocol. Colonies were incubated and selected on the medium (SD/-Leu/AbA) with the bait minimum AbA resistance for 3 days at 30 °C.

### 4.5. Dual-Luciferase Reporter Assay

The *pFaesELF3* region (−891/+571) was cloned into pGreen0800-LUC vector to generate the reporter plasmid pGreen0800-*pFaesELF3* with the primers Dual-pFaesELF3F and Dual-pFaesELF3R (Supplementary Table S1). The full-length ORFs of *FaesAP3_1* were cloned into the pGreenII 62-SK vector to generate the effector vectors with the primers Dual-FaesAP3_1F and Dual-FaesAP3_1R (Supplementary Table S1). The reporter vectors and two effectors were separately introduced into *Agrobacterium* strain GV3101 (pSoup). The effector and the reporter *Agrobacterium* cultures were mixed together and infiltrated into *N. benthamiana* leaves. Empty pGreenII 62-SK was cotransformed with the pGreen0800-*pFaesELF3* reporter as a negative control. Firefly luciferase and renilla luciferase were assayed at 3 days after being infiltrated using the Duo-Lite^TM^ Luciferase Assay System (Vazyme, Nanjing, China), following the manufacturer’s protocol, and the resulting luciferase signals were also captured by the Tanon 5200 image system (Tanon, Shanghai, China). Each combination was conducted with three biological replicates.

### 4.6. Cytomorphological Observation and Expression Analysis of FaesELF3

The LH floral buds fixed in FAA above were separately dehydrated in an ethanol series, cleared twice in xylene, infiltrated triple in molten paraffin, embedded into paraffin block, serially sectioned, and then sections were separately stained according to Liu et al. [41]. Each section was microscopically examined with a CAIKON RCK−40C microscope (CAIKON, Shanghai, China) and the photomicrographs were subsequently taken.

Total RNA of each sample was extracted with the EASY spin plant RNA Rapid Extraction Kit (Aidlab, Beijing, China). The first-strand cDNA was synthesized for qRT-PCR (quantitative real-time PCR) using the HiScript^®^ II Q RT SuperMix for qPCR kit (Vazyme, Nanjing, China), following the manufacturer’s protocol. *FaesELF3* expression was detected in seven organs (root, stem, juvenile leaf, tepal, stamen, pistil and 6-day-old fruit) of LH plants by qRT-PCR referring to Liu et al. [40], but with the primers qFaesELF3F and qFaesELF3R (Supplementary Table S1). In addition, *FaesELF3* expression was also detected in LH floral buds at sequential developmental stages with qRT-PCR mentioned above. The amplicons of *F. esculentum actin* gene (Genbank accession number: HQ398855.1) were amplified as the internal control with primers qFaesactinF and qFaesactinR. The experiments were repeated three times for each sample and the relative expression levels were measured according to Liu et al. [40], but with annealing at 60 °C.

### 4.7. VIGS Assay in Long-Homostyle Buckwheat

A 380 bp fragment of the *FaesELF3* cDNA was cloned into the TRV-mediated VIGS vectors with *Xba* I and *Sac* I restriction enzymes, as well as the primers TRV2-FaesELF3F and TRV2-FaesELF3R. In addition, a 423 bp fragment of the *FaesAP3_1* genomic DNA (Genbank accession number: MK335654.1) was cloned into the TRV-mediated VIGS vectors with *Xba* I and *Sac* I restriction enzymes and the primers TRV2-FaesAP3_1F and TRV2-FaesAP3_1R. Then, the TRV2-*FaesELF3*, TRV2-*FaesAP3_1,* TRV2 and TRV1 empty vector plasmids were separately introduced into *Agrobacterium* strain EHA105. The TRV-mediated VIGS vectors were infiltrated into leaves of the two-true-leaves stage seedlings of long-homostyle buckwheat mediated by *Agrobacterium tumefaciens* strain EHA105 with the method suggested by Liu [42]. All infected seedlings were cultivated in darkness for 24 h and then moved to a greenhouse with a temperature of 22 °C under short-day conditions. *FaesELF3* and *FaesAP3_1* expression levels were separately confirmed by qRT-PCR in flowers of *FaesELF3*-VIGS and *FaesAP3_1*-VIGS silencing plants.

## 5. Conclusions

The heteromorphic self-incompatibility (HSI) of buckwheat (*F. esculentum*) produces distylous flowers (pin and thrum) and results in low yield. Improving the yield stability of buckwheat requires a better understanding of the molecular basis of the HIS or developing a self-compatible (SC) common buckwheat line. Here, we have developed a self-compatible common buckwheat line with uniform long-homostyle (LH) flowers. Moreover, our previous studies identified two *AP3*-like genes, *FaesAP3_1* and *FaesAP3_2,* working together to specify stamen identity. Identification downstream genes of floral organ identity regulators are critical to revealing the molecular mechanisms underlying floral morphogenesis. However, a general regulatory pathway between floral organ identity genes and their downstream targets is still lacking in nonmodel species. In this study, we screened a direct downstream target gene, *FaesELF3*, of a stamen identity transcription factor, *FaesAP3_1*, in long-homostyle (LH) *F. esculentum* by using yeast one-hybrid (Y1H) and dual-luciferase reporter (DR) assays. Furthermore, *FaesAP3_1*-silenced plants had flowers with part stamens or anthers homeotically transformed into tepaloid structures. *FaesELF3*-silenced plants produced abnormal stamens consisting of short filaments and empty anthers (male sterile anther). All of these results suggested that the transcription factor (TF) FaesAP3_1 directly activates *FaesELF3* to regulate filament elongation and pollen grain development in LH buckwheat. Our data also suggested another stamen development pathway independent of *FaesAP3_1* remains in *F. esculentum.*

## Data Availability

All data generated or analyzed during this study are included in this published article.

## Supplementary Materials

The following supporting information can be downloaded at: https://www.mdpi.com/article/10.3390/ijms232214403/s1.

## References

[1] Huda M.N., Lu S., Jahan T., Ding M., Jha R., Zhang K., Zhang W., Georgiev M.I., Park S.U., Zhou M. (2021). Treasure from garden: Bioactive compounds of buckwheat. Food Chem..

[2] You W., Chen X., Zeng L., Ma Z., Liu Z. (2022). Characterization of *PISTILLATA*-like Genes and Their Promoters from the Distyly *Fagopyrum esculentum*. Plants.

[3] Matsui K., Yasui Y. (2020). Buckwheat Heteromorphic Self-Incompatibility: Genetics, Genomics and Application to Breeding. Breed. Sci..

[4] Zeng L., Zhang J., Wang X., Liu Z. (2021). Isolation and Characterization of *APETALA3* Orthologs and Promoters from the Distylous *Fagopyrum esculentum*. Plants.

[5] Mizuno N., Yasui Y. (2019). Gene flow signature in the S-allele region of cultivated buckwheat. BMC Plant Biol..

[6] Matsui K., Mizuno N., Ueno M., Takeshima R., Yasui Y. (2020). Development of co-dominant markers linked to a hemizygous region that is related to the self-compatibility locus (*S*) in buckwheat (*Fagopyrum esculentum*). Breed. Sci..

[7] Kappel C., Huu C.N., Lenhard M. (2017). A short story gets longer: Recent insights into the molecular basis of heterostyly. J. Exp. Bot..

[8] Anwer M.U., Davis A., Davis S.J., Quint M. (2020). Photoperiod sensing of the circadian clock is controlled by *EARLY FLOWERING 3* and *GIGANTEA*. Plant J..

[9] Zhu Z., Quint M., Anwer M.U. (2022). *Arabidopsis EARLY FLOWERING 3* controls temperature responsiveness of the circadian clock independently of the evening complex. J. Exp. Bot..

[10] Bu T., Lu S., Wang K., Dong L., Li S., Xie Q., Xu X., Cheng Q., Chen L., Fang C. (2021). A critical role of the soybean evening complex in the control of photoperiod sensitivity and adaptation. Proc. Natl. Acad. Sci. USA.

[11] Huang H., Gehan M.A., Huss S.E., Alvarez S., Lizarraga C., Gruebbling E.L., Gierer J., Naldrett M.J., Bindbeutel R.K., Evans B.S. (2017). Cross-species complementation reveals conserved functions for *EARLY FLOWERING 3* between monocots and dicots. Plant Direct.

[12] Ning Y., Shi X., Wang R., Fan J., Park C.H., Zhang C., Zhang T., Ouyang X., Li S., Wang G.L. (2015). *OsELF3-2*, an Ortholog of *Arabidopsis ELF3*, Interacts with the E3 Ligase *APIP6* and Negatively Regulates Immunity against *Magnaporthe oryzae* in Rice. Mol. Plant.

[13] Itoh H., Tanaka Y., Izawa T. (2019). Genetic Relationship Between Phytochromes and *OsELF3-1* Reveals the Mode of Regulation for the Suppression of Phytochrome Signaling in Rice. Plant Cell Physiol..

[14] De Folter S., Angenent G.C. (2006). Trans meets cis in MADS science. Trends Plant Sci..

[15] Melzer R., Kaufmann K., Theiben G. (2006). Missing Links: DNA-Binding and Target Gene Specificity of Floral Homeotic Proteins. Adv. Bot. Res..

[16] Schilling S., Pan S., Kennedy A., Melzer R. (2018). MADS-box genes and crop domestication: The jack of all traits. J. Exp. Bot..

[17] Fang Z.W., Li X.P., Li X.F., Liu Z.X. (2015). *FaesPI*, a *Fagopyrum esculentum PISTILLATA* Ortholog, Is Involved Only in Stamen Development. J. Plant Biol..

[18] Li L.Y., Fang Z.W., Li X.P., Liu Z.X. (2017). Isolation and Characterization of the C-class MADS-box Gene from the Distylous Pseudo-cereal *Fagopyrum esculentum*. J. Plant Biol..

[19] Wenkel S., Turck F., Singer K., Gissot L., Le Gourrierec J., Samach A., Coupland G. (2006). *CONSTANS* and the CCAAT Box Binding Complex Share a Functionally Important Domain and Interact to Regulate Flowering of *Arabidopsis*. Plant Cell.

[20] Dinh T.T., Girke T., Liu X., Yant L., Schmid M., Chen X. (2012). The Floral Homeotic Protein APETALA2 Recognizes and Acts through an AT-Rich Sequence Element. Development.

[21] Filichkin S.A., Leonard J.M., Monteros A., Liu P.P., Nonogaki H. (2004). A Novel Endo-*β-Mannanase* Gene in Tomato *LeMAN5* Is Associated with Anther and Pollen Development. Plant Physiol..

[22] Rogers H.J., Bate N., Combe J., Sullivan J., Sweetman J., Swan C., Lonsdale D.M., Twell D. (2001). Functional Analysis of cis-Regulatory Elements within the Promoter of the Tobacco Late Pollen Gene *g10*. Plant Mol. Biol..

[23] Solano R., Nieto C., Avila J., Canas L., Diaz I., Paz-Ares J. (1995). Dual DNA binding specificity of a petal epidermis-specific MYB transcription factor (MYB.Ph3) from *Petunia hybrida*. EMBO J..

[24] Kim S.Y., Chung H.J., Thomas T.L. (1997). Isolation of a novel class of bZIP transcription factors that interact with ABA-responsive and embryo-specification elements in the Dc3 promoter using a modified yeast one-hybrid system. Plant J..

[25] Zhang Z.L., Xie Z., Zou X., Casaretto J., Ho T.D., Shen Q.J. (2004). A Rice WRKY Gene Encodes a Transcriptional Repressor of the Gibberellin Signaling Pathway in Aleurone Cells. Plant Physiol..

[26] Chen P.W., Chiang C.M., Tseng T.H., Yu S.M. (2006). Interaction between Rice MYBGA and the Gibberellin Response Element Controls Tissue-Specific Sugar Sensitivity of alpha-Amylase Genes. Plant Cell.

[27] Mena M., Cejudo F.J., Isabel-Lamoneda I., Carbonero P. (2002). A Role for the DOF Transcription Factor BPBF in the Regulation of Gibberellin-Responsive Genes in Barley Aleurone. Plant Physiol..

[28] Baumann K., De Paolis A., Costantino P., Gualberti G. (1999). The DNA binding site of the Dof protein NtBBF1 is essential for tissue-specific and auxin-regulated expression of the *rolB* oncogene in plants. Plant Cell.

[29] Ross E.J., Stone J.M., Elowsky C.G., Arredondo-Peter R., Klucas R.V., Sarath G. (2004). Activation of the *Oryza sativa* non-symbiotic haemoglobin-2 promoter by the cytokinin-regulated transcription factor, ARR1. J. Exp. Bot..

[30] Agarwal M., Hao Y., Kapoor A., Dong C.H., Fujii H., Zheng X., Zhu J.K. (2006). A R2R3 Type MYB Transcription Factor Is Involved in the Cold Regulation of *CBF* Genes and in Acquired Freezing Tolerance. J. Biol. Chem..

[31] Simpson S.D., Nakashima K., Narusaka Y., Seki M., Shinozaki K., Yamaguchi-Shinozaki K. (2003). Two different novel cis-acting elements of *erd1*, a *clpA* homologous Arabidopsis gene function in induction by dehydration stress and dark-induced senescence. Plant J..

[32] Xue G.P. (2002). Characterisation of the DNA-binding profile of barley HvCBF1 using an enzymatic method for rapid, quantitative and high-throughput analysis of the DNA-binding activity. Nucleic Acids Res..

[33] Smyth D.R., Bowman J.L., Meyerowitz E.M. (1990). Early flower development in *Arabidopsis*. Plant Cell.

[34] Fang Z.W., Qi R., Li X.F., Liu Z.X. (2014). Ectopic expression of *FaesAP3*, a *Fagopyrum esculentum* (Polygonaceae) *AP3* orthologous gene rescues stamen development in an *Arabidopsis ap3* mutant. Gene.

[35] Wuest S.E., O’Maoileidigh D.S., Rae L., Kwasniewska K., Raganelli A., Hanczaryk K., Lohan A.J., Loftus B., Graciet E., Wellmer F. (2012). Molecular basis for the specification of floral organs by *APETALA3* and *PISTILLATA*. Proc. Natl. Acad. Sci. USA.

[36] Wang P., Liao H., Zhang W., Yu X., Zhang R., Shan H., Duan X., Yao X., Kong H. (2015). Flexibility in the structure of spiral flowers and its underlying mechanisms. Nat. Plants.

[37] Deveaux Y., Conde E., Silva N., Manicacci D., Le Guilloux M., Brunaud V., Belcram H., Joets J., Soubigou-Taconnat L., Delannoy E. (2021). Transcriptome Analysis Reveals Putative Target Genes of *APETALA3-3* During Early Floral Development in *Nigella damascena* L.. Front. Plant Sci..

[38] Higo K., Ugawa Y., Iwamoto M., Korenaga T. (1999). Plant cis-acting regulatory DNA elements (PLACE) database: 1999. Nucleic Acids Res..

[39] Clough S.J., Bent A.F. (1998). Floral Dip: A Simplified Method for *Agrobacterium*-Mediated Transformation of *Arabidopsis thaliana*. Plant J..

[40] Liu Z., Fei Y., Zhang K., Fang Z. (2019). Ectopic Expression of a *Fagopyrum esculentum APETALA1* Ortholog Only Rescues Sepal Development in *Arabidopsis Ap1* Mutant. Int. J. Mol. Sci..

[41] Liu Z.X., Xiong H.Y., Li L.Y., Fei Y.J. (2018). Functional Conservation of an *AGAMOUS* Orthologous Gene Controlling Reproductive Organ Development in the Gymnosperm Species *Taxus chinensis* var. mairei. J. Plant Biol..

[42] Liu Y., Schiff M., Dinesh-Kumar S.P. (2002). Virus-induced gene silencing in tomato. Plant J..

