# Supplementary material for: FaesAP3_1 Regulates the FaesELF3 Gene Involved in Filament-Length Determination of Long-Homostyle Fagopyrum esculentum
Source: Int J Mol Sci. 2022 Nov 19;23(22):14403. doi: 10.3390/ijms232214403 (PMC9694435; doi:10.3390/ijms232214403)
Supplement: Supplementary file 1 [file ijms-23-14403-s001.zip › ijms-2015944-supplementary.pdf]

Supplementary Materials

Table S1. Primers used in this study.

Primer name	Primers sequences (5'-3')
3RELF3GSP	GGGATGAGTTTATCCGACAATTC
5RELF3GSP1	TGAATGTGATGCCAGAG
5RELF3GSP2	GCTACTCCCCTGACTTGCTCCACTATGCTGAT
5RELF3GSP3	TGGGTTGTGCCGATTTGAAGG
DpELF3SP1	TCCCCTGACTTGCTCCACTATGC
DpELF3SP2	GCCGATTTGAAGGGATAGTAAGTTGC
DpELF3SP3	CCATGGACTTCTCCTCGTATTTAC
DpELF3SP4	GATGGACACAAACCCAATAGACCCA
DpELF3SP5	GGGGTTAGAAAGAAGATTGGGGTTG
DpELF3SP6	AAGGAAAGTGCTATGAGTTGTGGGC
TpFaesELF3F1	ATGATTACGAATTCGAGCTCCGATCAACTAGTTATTTTT
TpFaesELF3F2	ATGATTACGAATTCGAGCTCCAATCAAACTCTTAATAATCGAA
TpFaesELF3F3	ATGATTACGAATTCGAGCTCCTATTCCGTATCTTGTAGTTG
TpFaesELF3R	TCTGCAGGTCGACTCTAGACTCTTATTTCAAAATTTTCAACC
Y1HpFaesELF3F	AAGCTTGAATTCGAGCTCTCCATTTAGGGAAACAGGA
Y1HpFaesELF3R	ACATGCCTCGAGGTCGACCTCTTATTTCAAAATTTTC
Y1HFaesAP3_1F	ATGGAGGCCAGTGAATTCTGCTAAGAACAACAAGTAACA
Y1HFaesAP3_1R	ATTCATCTGCAGCTCGAGCTCGAACACATGAACACAATGAG
Y1HFaesAP3_2F	ATGGAGGCCAGTGAATTCATGTTACAGAGCCAATTACGA
Y1HFaesAP3_2R	ATTCATCTGCAGCTCGAGCTCCATATACAATCGGAGACAC
Y1HFaesPIF	ATGGAGGCCAGTGAATTCATGGGGAGAGGGAAGATTG
Y1HFaesPIR	ATTCATCTGCAGCTCGAGCTCTAGGGTTTTTGCTCTTGCTAC
Y1HFaesAGF	ATGGAGGCCAGTGAATTCTCTTTTAGTTTCTTCTCCCAG
Y1HFaesAGR	ATTCATCTGCAGCTCGAGCTCTACAACACAGCTTCTCAAC
Dual-pFaesELF3F	GGGCCCCCCCTCGAGGTCGACCAATCAAACTCTTAATAATCG
Dual-pFaesELF3R	CAGGAATTCGATATCAAGCTTCTCTTATTTCAAAATTTTCAACC
Dual-FaesAP3_1F	CGCGGTGGCGGCCGCTCTAGATGCTAAGAACAACAAGTAACA
Dual-FaesAP3_1R	AAGCTTGATATCGAATTCGAACACATGAACACAATGAG
qFaesELF3F	CGGAAAGAGGAGGGCCAAGAG
qFaesELF3R	GACGCGTATTTCCTGGGTTGT
qFaesAP3_1F	AACCAACAGACAAGTTACTTATT
qFaesAP3_1R	ACATCCTGCCAGAACTCC
qFaesactinF	ACCTTGCTGGACGTGACCTTAC
qFaesactinR	CCATCAGGAAGCTCATAGTTC

TRV2-FaesELF3F	GTTACCGAATTCTCTAGAGATGAGTTTATCCGACAATTC
TRV2-FaesELF3R	CTCGAGACGCGTGAGCTCTGTCTTTGAAGTCTATCAGCT
TRV2-FaesAP3_1F	GTTACCGAATTCTCTAGACACCTTCCTTACTTCATCTCC
TRV2-FaesAP3_1R	CTCGAGACGCGTGAGCTCCTTCATGCATTATACAGGACA

Table S2. Information of sequences selected for alignments and phylogenetic analyses from NCBI GenBank.

Taxon and species	Protein name	Accession numbers
Poaceae		
Oryza sativa	OsELF3_1	CAA08804.1
	OsELF3_2	CAA08802.1
Zea mays	ZmELF3_1	NP_001169653.1
	ZmELF3_2	PWZ32646.1
Triticum aestivum	TaELF3_1DL	ABL11477.1
Hordeum vulgare	HvELF3	AEZ53982.1
Brachypodium distachyon	BdELF3	XP_003567779.1
Setaria viridis	SvELF3a	TKW15056.1
	SvELF3b	TKW25484.1
Ranunculaceae		
Paeonia suffruticosa	PsELF3	QJP03923.1
Fabaceae		
Cicer arietinum	CaELF3a	XP_004501482.1
	CaELF3b	XP_004498926.1
Lens culinaris	LcELF3	AFZ78447.1
Pisum sativum	PsELF3a	AFR60580.1
Glycine max	GmELF3a	AQX44338.1
	GmELF3b_1	AQX44344.1
	GmELF3b_2	XP_003550290.1
Malvaceae		
Gossypium hirsutum	GhELF3	XP_016744799.2
Brassicaceae		
Arabidopsis thaliana	AtELF3	AAX23847.1
Brassica rapa	BrELF3	AEB33726.1
Polygonaceae		
Fagopyrum esculentum	S-ELF3	BAL45488.1
	FaesELF3	OP572281.1

Aizoaceae		
Mesembryanthemum crystallinum	McELF3	AAQ73529.1

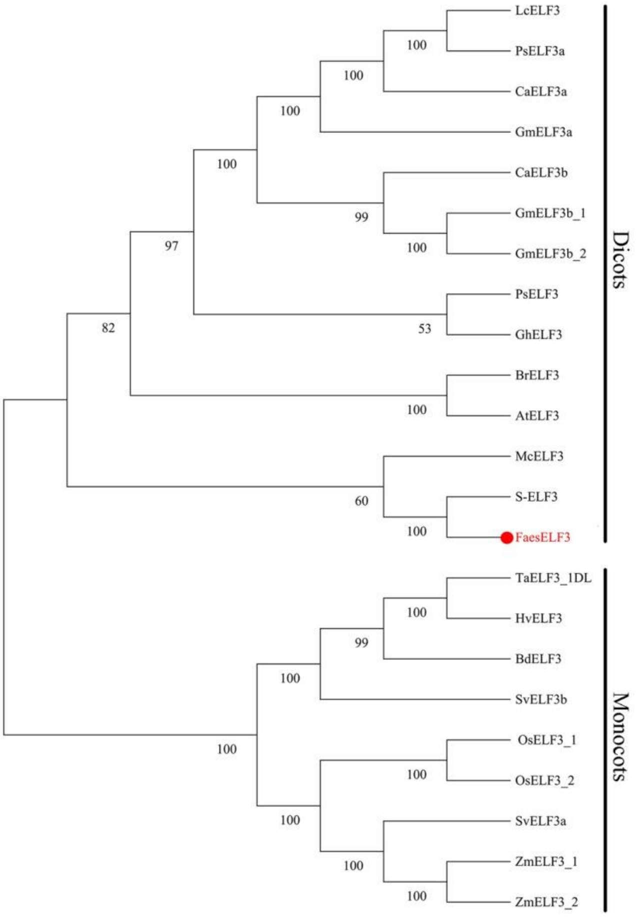

Supplementary Figure S1. Phylogenetic tree of FaesELF3 and other ELF3 homologous proteins from different species.

> pFaesELF3

-1278 CGATCAACTA G**TTATTT**TTG CTACT**CAAT**A AGCTTAGGTT ATCGATAAAC TTTATATGCG

TATABOX5 CAATBOX1

-1218 ACCAGTAA**AC ACAAG**GTTTC CAA**ACGT**ACC CTCAAACAT**C AAT**TTCATAA AACACACTCA

DPBFCOREDCDC3 ACGTATERD1 CAATBOX1

-1158 AATTCCACAG CTTTCAAAAA TTATATTTGG **TATATAA**ACA CTAATTAATA TCACAAACA**C**

TATABOX4

-1098 **AATATAATG**T T**ACACACGAG** AGGTGTTCGC TCCCAAATTA GTATAAAAAA TCAGT**GTGA**A

CArG-box DPBFCOREDCDC3 GTGANTG10

-1038 AATGAATATT TTTTTTATCT TATA**CCTTTT** TTCGT**TATAT AA**ATAAAGGT TGGTGGAATA

PYRIMIDINEBOXOSRAMY1A TATABOX4

-978 CATAATAACA TAAGTGCAAG ACCCTCGACA TCTATTTTTT ATACATTAAA TTTATCCTTA

-918 AAAATTGAAA TATTT**CAAT**G CAAATAT**CAA T**CAAACTCTT AATAATCGAA TC**AACAAA**GA

CAATBOX1 CAATBOX1 AACAAA motif

-858 ACTCGTACTT AAACCGAATC TCTTGTAAAC **CGATT**AGTTT AGGTTGAAAA **GTGA**CACAAG

ARR1AT GTGANTG10

-798 CTA**AACAAA**A TAAATCGTCA TAAATTGCAA AATAGTAAAT TCCAGAT**CAA T**TTTTA**CAAT**

AACAAA motif CAATBOX1 CAATBOX1

-738 TATCAAAAAG TTGGACGGCA AAAAATACAC TTAAAAGTTA AATTGCATTA TGAGTTGAAG

-678 ACTTGAAG**CA AT**AATCTTGT TTTA**TGAC**AA GTGTAAGCAA AAGCGTCATT ACTTTTCCCA

CAATBOX1 WRKY71OS

-618 TAAATTATGT TCACTGGATA AGGTAAAATA TACTACAACA TCACTAACAT A**GTGA**CATAT

GTGANTG10

-558 CCATTATGAT ATCTTATTCT CTTCAACTAC TCTT**CAAT**TA CCCCGAAGAT AATCAT**CAAT**

CAATBOX1 CAATBOX1

-498 TAGGCAATTA ATCAACAATT AAGCACACAA CAGTAGACCA GTAGCTTAAG TTTACCCAAC

-438 CAAAGTCTTC AAAGGTAAGA AGAAGCGTGG AAAATCTGCA G**CCAAT**GATA AAACGGCTAA

CCAATBOX1

-378 AG**ATCGAC**TG ATTTCATCAC CATAGATTGT TGGGAAGGA**A GAAA**TC**GTGA** AATATCGTAA

CBFHV POLLEN1LELAT52 GTGANTG10

-318 AAGGTACTAG TATTGTATTG ACAACAAGCT CGTAGCTC**TA TCCA**AATCGT GGCCCACAAC

TATCCAOSAMY

-258 CTCATAGCAC TTTC**CCTTTT** TTCC**CCTTTT** CATCGAATCC **CCAAT**TTCCC CTTCAACC**CC**

PYRIMIDINEBOXOSRAMY1A CCAATBOX1

-198 **AAT**CTTCTTT CTAACCCCTT TGAATCTGTT CTCGTTCTCT TTCTGGGTCT ATTGGGTTTG

CCAATBOX1

-138 TGTCCATCTA CTTACCTATC TACAACAAGC TTTCCTGGAT TCACTCGTGC AA**ACTTTA**TT

NTBBF1ARROLB

-78 GGTC**CATTTG** ATATTTTTAC TTCCTTGCTC CTGAGCTTTT GTAACTCCAT TTAGGGAAAC

MYCCONSENSUSAT +1

-18 AGGAAAATCT AGCATGAGAT TGTGTTTTTA GAGTTGAGGT TG**CCTTTT**CT CGTA**CAGTTA**

PYRIMIDINEBOXOSRAMY1A MYBCORE

+43 GGTGGACTTC GCTTTCGCAG **GTGA**GAAGAT CGGAGGATCT GGGATTAGGG **TTTGTT**TGAT

GTGANTG10 TTTGTT motif

+103 TTTGGATAAT TATTCCTGGT TAGT**CAAT**TC TATGATCTGA AGTTGCCTAG TTATGCTATC

CAATBOX1

+163 TTGTTGTTGG GTTCTGGTGT GTTTAATTTT G**AACAAA**TTG TAGTTGGTAA GT**CAAT**GCTA

AACAAA motif CAATBOX1

+223 TGCTCTGAAG TTG**CTTATTT AAG**CTATTCC GTATCTTGTA GTTGGGTTCT CTAGCTGAGA

CArG-box

+283 GCATAAGATC TTAATTAGGT ATTTATTGGG TTCTGGG**TTT GTT**TGATTTT GTGTTTTTGG

TTTGTT motif

+343 AACTTGCTGG GGTCATAGTA GCT**AGAAA**TG GAATTGTTTG CTTCTGATTT TATGTACAAG

POLLEN1LELAT52

+403 CGTGTGTGC**A GAAA**AGCTTA CCTTGCACTT TTGTAATGTG TTCTTTGCAG GAGTTGCTTG

POLLEN1LELAT52

+463 ATTGGGAGTT CTGGGGATGA GTTTATCCGA **CAAT**T**CAAGT G**GAGGAGCTT TTATGGAACC

CAATBOX1 MYCCONSENSUSAT

+523 TGTGCC**TTTG TT**GGATTGTT AAGAGAGGTT GAAAATTTTG AAATAAGAG**ATG**

TTTGTT motif

Supplementary Figure S2. ***pFaesELF3*** **promoter sequence.** The transcription start site (+1) is in bold and boxed. The start codon ATG is in bold and boxed. Putative cis-acting regulatory elements are in bold and underlined.
